# Red Cell Indices–Based Morphological Patterns of Anemia and Associated Factors Among Adults With Diabetes Mellitus: A Cross‐Sectional Study at Mubende Regional Referral Hospital, Uganda

**DOI:** 10.1155/anem/4849833

**Published:** 2026-05-21

**Authors:** Feisal Dahir Kahie, Amon Banturaki, Marie Pascaline Sabine Ishimwe, Stella Nabirye, Abshir M. Hirsi, Venance Emmanuel Mswelo, Awil Abdulkadir Abdi, Elias Joseph Xwatsal, Hanan Asad Hassan, Hamdi Mohamed Yusuf, Emmanuel Ifeanyi Obeagu, Theoneste Hakizimana

**Affiliations:** ^1^ Department of Internal Medicine, Kampala International University, Ishaka, Uganda, kiu.ac.ug; ^2^ Department of Pediatrics and Child Health, Kampala International University, Ishaka, Uganda, kiu.ac.ug; ^3^ Department of Medical Laboratory Science, Kampala International University, Ishaka, Uganda, kiu.ac.ug; ^4^ Department of Obstetrics and Gynecology, Kampala International University, Ishaka, Uganda, kiu.ac.ug

**Keywords:** anemia, diabetes mellitus, macrocytic anemia, microcytic anemia, morphological patterns, normocytic anemia, Uganda

## Abstract

**Background:**

Diabetes mellitus (DM) is a major public health concern, and anemia is a common but often overlooked complication that can worsen fatigue, quality of life, and clinical outcomes. In Uganda, where diabetes is increasing, evidence on red cell indices–based anemia patterns among adults with DM remains limited. We determined the prevalence, anemia subtypes, and associated factors among adults with DM at Mubende Regional Referral Hospital (MRRH).

**Methods:**

We conducted a hospital‐based cross‐sectional study among consecutively recruited adults with DM (*n* = 246) at MRRH. Sociodemographic and clinical data were collected using a structured questionnaire and physical examination. Hemoglobin was measured using an automated hematology analyzer. Anemia was defined using the World Health Organization cutoffs, and anemia subtypes were classified by mean corpuscular volume (microcytic, normocytic, and macrocytic). Data were analyzed in STATA 15.1. Logistic regression was used to identify factors independently associated with anemia; *p* < 0.05 was considered statistically significant.

**Results:**

The prevalence of anemia was 28.0% (69/246; 95% CI: 22.8%–34.3%). Among anemic participants, normocytic anemia was most common (76.81%; 95% CI: 66.85%–86.77%), followed by microcytic anemia (13.04%; 95% CI: 5.10%–20.98%) and macrocytic anemia (10.14%; 95% CI: 3.02%–17.27%). In multivariable analysis, older age (> 60 years) (AOR = 5.17, *p* = 0.022), neuropathy (AOR = 4.97, *p* < 0.001), diabetes duration > 5 years (AOR = 2.25, *p* = 0.045), obesity (AOR = 5.94, *p* = 0.049), and poor glycemic control (AOR = 2.96, *p* = 0.002) were independently associated with anemia.

**Conclusion:**

Anemia was common among adults with DM at MRRH, with normocytic anemia as the predominant red cell indices–based subtype. Routine anemia screening should be integrated into diabetes care, with further etiologic evaluation (e.g., iron studies, vitamin B12/folate, and renal markers) where feasible.

## 1. Introduction

Diabetes mellitus (DM) is a chronic metabolic disorder characterized by persistent hyperglycemia due to impaired insulin secretion, insulin action, or both, with downstream effects on carbohydrate, protein, and fat metabolism [1, 2]. Type 2 diabetes mellitus (T2DM), the predominant form worldwide, is driven largely by insulin resistance and requires care that extends beyond glucose control to prevention and management of multisystem complications [3–5]. In parallel, anemia remains a major global health problem and disproportionately affects African populations, contributing to morbidity and reduced functional capacity [6–8].

Anemia is more common in people with DM than in those without DM and is linked to worse microvascular disease and overall morbidity [9]. The pathways are multifactorial, including inflammation, nutritional deficiencies (iron, vitamin B12, and folate), medication effects, and diabetes‐related organ dysfunction [10–12]. Epidemiologically, a meta‐analysis reported a pooled anemia prevalence of 27.0% among adults with T2DM [13], while pooled evidence from Africa suggests an even higher burden, particularly among patients with diabetic foot ulcers and poor glycemic control [13]. Individual studies show wide variability, from 8.06% in one Ethiopian cohort to 27.5% in another, and have linked anemia to factors such as older age, poor glycemic control, longer diabetes duration, obesity, hypertension, microvascular complications, nephropathy [9], and kidney function markers [14, 15].

In Uganda, diabetes is increasing and often underdiagnosed, and anemia remains a persistent public health challenge [10, 16]. Yet anemia in adults with diabetes is under‐recognized and not routinely screened for, partly because symptoms overlap with those of chronic disease [9, 15]. At Mubende Regional Referral Hospital (MRRH), adults with diabetes are managed through the outpatient clinic, but routine anemia screening is not integrated into standard care (MRRH,2023, Unpublished). This study therefore aimed to determine the prevalence of anemia, describe red cell indices–based morphological patterns, and identify factors associated with anemia among adults with diabetes attending MRRH.

## 2. Materials and Methods

This was a hospital‐based cross‐sectional study using quantitative methods. It was conducted at the Diabetic Outpatient Clinic of MRRH in Mubende Town, Central Uganda, over a 3‐month period (October 2024 to January 2025), to determine the prevalence of anemia, red cell indices–based morphological patterns, and associated factors among adults with DM. MRRH is the referral hospital for Mubende District and surrounding districts and provides free general medical services. It also serves as a training site for medical graduates completing a 1‐year internship under the supervision of qualified physicians and specialists. As of July 2020, the hospital’s catchment population was estimated at about 610,600 residents. The hospital has an estimated 240‐bed capacity and offers both inpatient and outpatient services across multiple specialties, including internal medicine, surgery, pediatrics, emergency care, and obstetrics and gynecology. It is affiliated with Kampala International University (KIU) as a teaching facility.

The internal medicine department runs daily general outpatient services and a specialized diabetic clinic every Thursday. The hospital laboratory is staffed by qualified personnel and supports diabetes care through routine investigations including blood glucose and glycated hemoglobin (HbA1c). These laboratory services also enabled measurement of hemoglobin concentration and red blood cell indices for anemia assessment and morphological classification.

### 2.1. Study Population

The study included adult patients (≥ 18 years) with a confirmed diagnosis of DM attending the Diabetic Outpatient Clinic at MRRH during the study period, who provided written informed consent. Participants were excluded if they had known hematologic disorders (e.g., sickle cell anemia), chronic conditions that could independently cause anemia (e.g., systemic lupus erythematosus or rheumatoid arthritis), a history of chronic kidney disease (CKD), recent blood loss within the past week, a blood transfusion in the preceding 3 months, or pregnancy, to minimize confounding when estimating anemia prevalence and evaluating associated factors. We excluded participants with documented CKD in the clinical record and those receiving renal replacement therapy. Renal function tests (e.g., serum creatinine/eGFR) and albuminuria were not measured as part of this study; therefore, subclinical kidney dysfunction could not be evaluated.

### 2.2. Sample Size Calculation

The sample size for morphological pattern of anemia in diabetic patients was computed using Kish Leslie’s (1965)​ equation shown below:

(1) n=Zα2p 1−pd2,

where *Z* *α* = 1.96; *p* = 0.2 since 20% of DM patients were anemic in a study done in Ethiopia [15]; and *d* = 0.05. On substituting, *n* = 246.

The minimum estimated sample size, which is 246, was considered.

### 2.3. Sampling Techniques

A consecutive sampling approach was employed. On each diabetic clinic day (every Thursday) during the study period, all adult patients with a confirmed diagnosis of DM who attended the Diabetic Outpatient Clinic at MRRH were screened for eligibility. Those who met the inclusion criteria and provided written informed consent were enrolled consecutively. Recruitment continued clinic by clinic until the required sample size of 246 participants was achieved.

### 2.4. Data Collection Procedure

Following written informed consent, adult patients with DM were consecutively recruited from the Diabetic Outpatient Clinic at MRRH during the study period (October 2024–January 2025). Participants were enrolled after consenting in either English or Luganda. Data were collected by the principal investigator with support from trained research assistants using a structured questionnaire capturing sociodemographic, behavioral, and medical characteristics. A brief physical examination was performed, including assessment for clinical signs suggestive of neuropathy. Anthropometric measurements (height and weight) were obtained using standardized procedures, and body mass index (BMI) was computed and recorded. Venous blood samples were then collected for laboratory investigations, including HbA1c analysis using Siemens ADVIA series analyzers. Hemoglobin concentration was measured using an automated hematology analyzer with routine quality control checks. Throughout data collection, facility safety and infection‐prevention measures (including Ebola and COVID‐19 precautions) were adhered to.

### 2.5. Assessment Tool and Diagnostic Criteria

Anemia was defined using the World Health Organization hemoglobin cutoffs as hemoglobin < 13.0 g/dL in men and < 12.0 g/dL in women. Morphological patterns of anemia were classified primarily using mean corpuscular volume (MCV) generated by the automated hematology analyzer and categorized as microcytic, normocytic, or macrocytic. Although mean corpuscular hemoglobin and mean corpuscular hemoglobin concentration were also generated by the analyzer, they were not used as the primary basis for classification. Peripheral blood smear examination was not performed. Glycemic control was defined using HbA1c, with poor glycemic control defined as HbA1c ≥ 7.0% and good glycemic control defined as HbA1c < 7.0%. Neuropathy was assessed clinically during the study examination using participants’ symptoms and physical examination findings suggestive of peripheral neuropathy, as documented on the study tool.

### 2.6. Study Variables

The primary outcome was anemia status (anemia vs. no anemia), while a secondary outcome was the morphological pattern of anemia among anemic participants (normocytic, microcytic, or macrocytic). Independent variables included sociodemographic, behavioral, clinical, and laboratory factors. Anemia was defined using WHO hemoglobin cutoffs: < 13.0 g/dL in men and < 12.0 g/dL in nonpregnant women [17]. Neuropathy was assessed clinically by trained study personnel using symptoms and examination findings suggestive of peripheral neuropathy, without nerve conduction studies or a validated scoring tool. Glycemic control was based on HbA1c, with poor control defined as HbA1c ≥ 7.0% and good control as HbA1c < 7.0%. Morphological classification of anemia was based primarily on MCV from the automated hematology analyzer; although MCH and MCHC were generated, they were not used as the main basis for classification, and peripheral blood smear was not performed. The study included adults aged ≥ 18 years with a confirmed diagnosis of DM attending the Diabetic Outpatient Clinic, regardless of diabetes type. Diabetes duration was categorized as < 5 years or > 5 years, and medication type as single oral therapy, combination oral therapy, insulin alone, or oral agents plus insulin. BMI was classified using standard WHO adult categories, alcohol use was self‐reported as yes or no, and hypertension status was defined from documented history and/or current antihypertensive treatment.

### 2.7. Quality Control

To ensure tool validity, questionnaire content validity was assessed using the Content Validity Index (CVI) based on feedback from two physicians at MRRH; a CVI ≥ 0.80 was considered acceptable. The questionnaire was pretested prior to the main study. Reliability was assessed through a pilot (ten pretests), and internal consistency was evaluated using Cronbach’s alpha, with α > 0.80 considered acceptable. For laboratory quality, internal quality control procedures and strict adherence to standard operating procedures were maintained, and the laboratory processes followed recognized accreditation standards (SANAS). Research assistants (qualified nurses and medical officers) were trained 1 week before data collection, study instruments were routinely calibrated, and data collection was supervised by physicians and the principal investigator to ensure adherence to protocol.

### 2.8. Data Management and Analysis

Data were entered, checked for completeness, and cleaned in Microsoft Excel 2021, then exported to Stata Version 15.1 (StataCorp, College Station, TX, USA) for analysis. Continuous variables (e.g., age, hemoglobin level, HbA1c, and other laboratory/clinical measurements where applicable) were summarized using means and standard deviations (or medians and interquartile ranges when not normally distributed). Categorical variables including sociodemographic and clinical characteristics (e.g., sex, education level, residence, BMI category, income category, physical activity, smoking, alcohol use, diabetes duration, medication type, hypertension status, glycemic control category, and neuropathy status) were summarized using frequencies and percentages.

The prevalence of anemia was calculated as the proportion of participants who met the WHO hemoglobin cutoffs for anemia, and results were presented as percentages (with 95% confidence intervals [CIs]). Among participants with anemia, morphological patterns of anemia were classified using red cell indices into normocytic, microcytic, and macrocytic categories, and the distribution of these patterns was summarized as percentages and displayed using a bar chart. To explore factors associated with anemia, a binary outcome variable was generated (1 = anemia; 0 = no anemia). Bivariate logistic regression was used to assess associations between each independent variable and anemia. Variables with *p* ≤ 0.20 at bivariate analysis and/or those considered biologically plausible were included in a multivariable logistic regression model to identify independent predictors. Crude odds ratios (cORs) and adjusted odds ratios (aORs) with corresponding 95% CIs were reported, and statistical significance was set at *p* < 0.05.

Multicollinearity was assessed using variance inflation factors (VIFs) (VIF < 2.0 considered acceptable). Model fit/calibration was assessed using the Hosmer–Lemeshow goodness‐of‐fit test (*p* > 0.05), alongside standard classification diagnostics where applicable. All analyses were two‐sided with α = 0.05, and results were reported as ORs with 95% CIs and *p* values in line with journal requirements.

### 2.9. Human Ethics and Consent to Participate

This study was approved by KIU‐REC (KIU‐2024‐399) and registered with UNCST. All participants were ≥ 18 years and gave written informed consent. We followed STROBE guidelines for cross‐sectional studies and SAMPL recommendations for statistical reporting. All participants provided written informed consent after being fully informed about the study’s purpose, procedures, and rights. Participation was voluntary, and only individuals aged 18 years or older were enrolled, each providing consent personally.

## 3. Results

**FIGURE 1:**
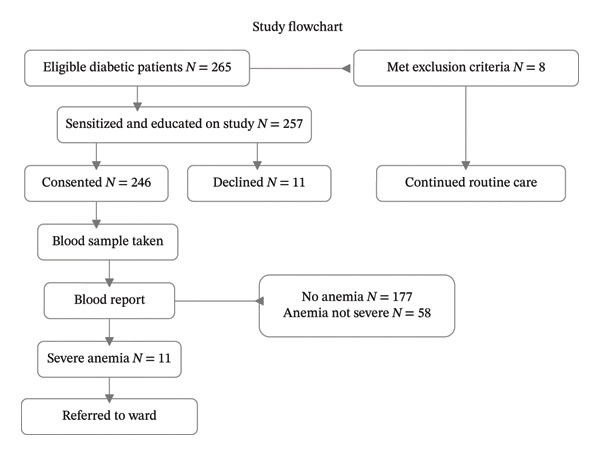
Study flowchart.

A total of 265 adults with DM attending the Diabetic Outpatient Clinic were screened for participation. Of these, 257 met the eligibility criteria, while 8 were excluded at eligibility assessment. Among the 257 eligible participants, 246 consented and were enrolled in the study, whereas 11 were not enrolled. Complete data were collected from all 246 enrolled participants; therefore, all 246 were included in the final analysis (Figure 1).

### 3.1. Basic Characteristics of the Study Participants

A total of 246 participants were enrolled in this study. Most were female (62.2%), and the largest age group was 41–50 years (27.6%). Just over half were married (51.6%), the majority had no formal education (69.1%), and most lived in rural areas (70.3%). In terms of nutritional status, most participants had a normal BMI (73.6%), while 12.6% were underweight and 3.3% were obese. Over half reported engaging in physical activity (54.1%), though smoking (13.8%) and alcohol consumption (37.4%) were also reported. Clinically, diabetes duration was predominantly less than 5 years (76.8%). Half of the participants were on a single oral medication (50.0%), while 16.3% were on insulin. More than a third had poor glycemic control (34.9%), and nearly half had neuropathy (45.9%) (Table 1).

**TABLE 1:** Sociodemographic and clinical characteristics of participants (*N* = 246).

Characteristic	Frequency (*n*)	Percentage (%)
Age group (years)		
18–29	36	14.6
30–40	59	24
41–50	68	27.6
51–60	51	20.7
> 60	32	13
Sex		
Male	93	37.8
Female	153	62.2
Marital status		
Single	37	15
Married	127	51.6
Divorced	67	27.2
Separated	15	6.1
Education level		
Nonformal	170	69.1
Formal	76	30.9
Residence		
Urban	73	29.7
Rural	173	70.3
BMI		
Underweight	31	12.6
Normal	181	73.6
Overweight	26	10.6
Obese	8	3.3
Monthly income (UGX)		
> 200,000 UGX	73	29.7
< 200,000 UGX	173	70.3
Physical activity		
Yes	133	54.1
No	113	45.9
Smoking		
Yes	34	13.8
No	212	86.2
Alcohol consumption		
Yes	92	37.4
No	154	62.6
Diabetes duration		
< 5 years	189	76.8
> 5 years	57	23.2
Medication type		
Single oral	123	50
Combination oral	65	26.4
Insulin	40	16.3
Oral + insulin	18	7.3
Hypertension		
Yes	95	38.6
No	151	61.4
Glycemic control		
Poor	86	34.9
Good	160	65.1
Neuropathy		
Yes	113	45.9
No	133	54.1

### 3.2. Prevalence of Anemia Among Adults With DM

**FIGURE 2:**
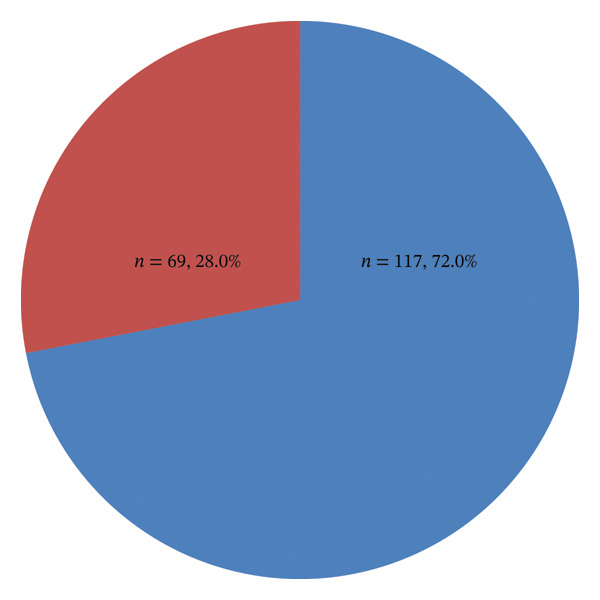
Prevalence of anemia among adults with diabetes mellitus. Red: anemia; blue: no anemia.

**FIGURE 3:**
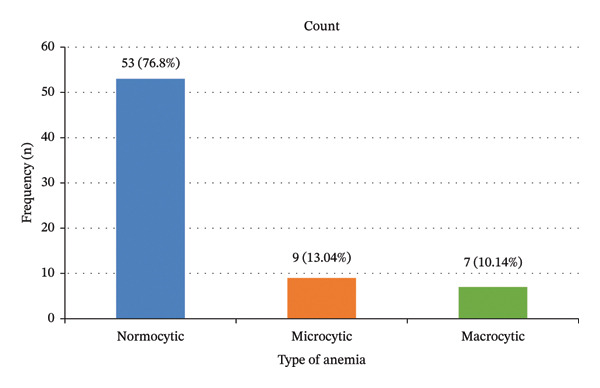
Morphological characteristics of anemia among diabetic patients (*N* = 69).

Overall, 69 of 246 (28.0%; 95% CI: 22.4–33.7) participants had anemia, while 177 of 246 (72.0%) did not (Figure 2). By morphological type, normocytic anemia was most common, affecting 21.5% (53/246) of participants (i.e., 76.8% (53/69) of all anemia cases). Microcytic anemia occurred in 3.7% (9/246) of participants (13.0% (9/69) of anemia cases), and macrocytic anemia occurred in 2.8% (7/246) (10.1% (7/69) of anemia cases) (Figure 3).

### 3.3. Factors Associated With Anemia: Bivariate Analysis

Factors with *p* < 0.2 included age, sex, rural residence, underweight/obesity, diabetes duration > 5 years, combination oral therapy, poor glycemic control, and neuropathy (Table 2). These were included in the multivariate model for further analysis. In multivariate analysis, older age (> 60 years), neuropathy, longer diabetes duration (> 5 years), obesity, and poor glycemic control remained significant predictors of anemia. Patients over 60 years had five times higher odds of anemia, while those with neuropathy had five times increased odds. Diabetes duration > 5 years doubled the odds, and obese individuals had six times higher odds of anemia. Additionally, poor glycemic control nearly tripled the likelihood of anemia (Table 3).

**TABLE 2:** Bivariate analysis of factors associated with anemia (*N* = 246).

Characteristic	No anemia *n* (%)	Anemia *n* (%)	COR (95% CI)	*p* value
Age group (years)				
18–29	32 (18.1)	4 (5.8)	1.00 (Ref)	—
30–40	44 (24.9)	15 (21.7)	2.73 (0.83–8.99)	**0.099**
41–50	51 (28.8)	17 (24.6)	2.67 (0.82–8.64)	**0.102**
51–60	34 (19.2)	17 (24.6)	4.00 (1.22–13.17)	**0.023**
> 60	16 (9.0)	16 (23.2)	8.00 (2.29–27.90)	**0.001**
Sex				
Male	69 (39.0)	24 (34.8)	1.00 (Ref)	—
Female	108 (61.0)	45 (65.2)	1.20 (0.67–2.14)	0.540
Marital status				
Single	23 (13.0)	14 (20.3)	1.48 (0.69–3.19)	0.316
Married	90 (50.8)	37 (53.6)	1.00 (Ref)	—
Divorced	51 (28.8)	16 (23.2)	0.76 (0.39–1.51)	0.436
Separated	13 (7.3)	2 (2.9)	0.37 (0.08–1.74)	0.210
Education level				
Nonformal	119 (67.2)	51 (73.9)	1.38 (0.74–2.57)	0.309
Formal	58 (32.8)	18 (26.1)	1.00 (Ref)	—
Residence				
Urban	57 (32.2)	16 (23.2)	1.00 (Ref)	—
Rural	120 (67.8)	53 (76.8)	1.57 (0.83–2.99)	**0.166**
BMI				
Underweight	18 (10.2)	13 (18.8)	2.18 (0.99–4.80)	**0.052**
Normal	136 (76.8)	45 (65.2)	1.00 (Ref)	—
Overweight	20 (11.3)	6 (8.7)	0.91 (0.34–2.40)	0.843
Obese	3 (1.7)	5 (7.3)	5.04 (1.16–21.92)	**0.031**
Monthly income (UGX)				
> 200,000 UGX	49 (27.7)	24 (34.8)	1.39 (0.77–2.53)	0.275
≤ 200,000 UGX	128 (72.3)	45 (65.2)	1.00 (Ref)	—
Physical activity				
Yes	97 (54.8)	36 (52.2)	1.00 (Ref)	—
No	80 (45.2)	33 (47.8)	1.11 (0.64–1.94)	0.710
Smoking				
Yes	26 (14.7)	8 (11.6)	0.76 (0.33–1.78)	0.528
No	151 (85.3)	61 (88.4)	1.00 (Ref)	—
Alcohol consumption				
Yes	62 (35.0)	30 (43.5)	1.43 (0.81–2.52)	0.220
No	115 (65.0)	39 (56.5)	1.00 (Ref)	—
Diabetes duration				
< 5 years	146 (82.5)	43 (62.3)	1.00 (Ref)	—
> 5 years	31 (17.5)	26 (37.7)	2.85 (1.53–5.31)	**0.001**
Medication type				
Single oral	93 (52.5)	30 (43.5)	1.00 (Ref)	—
Combination oral	40 (22.6)	25 (36.2)	1.94 (1.01–3.70)	**0.045**
Insulin	32 (18.1)	8 (11.6)	0.77 (0.32–1.86)	0.569
Oral + insulin	12 (6.8)	6 (8.7)	1.55 (0.54–4.49)	0.419
Hypertension				
Yes	67 (37.9)	28 (40.6)	1.12 (0.64–1.98)	0.693
No	110 (62.1)	41 (59.4)	1.00 (Ref)	—
Glycemic control				
Poor	52 (29.4)	34 (49.3)	2.34 (1.32–4.14)	**0.004**
Good	125 (70.6)	35 (50.7)	1.00 (Ref)	—
Neuropathy				
Yes	66 (37.3)	47 (68.1)	3.59 (1.99–6.49)	**< 0.001**
No	111 (62.7)	22 (31.9)	1.00 (Ref)	—

*Note:* Ref, reference category. Bold is *p* < 0.2.

Abbreviations: aOR, adjusted odds ratio; cOR, crude odds ratio.

**TABLE 3:** Multivariate analysis of factors associated with anemia (*N* = 246).

Characteristic	cOR (95% CI)	aOR (95% CI)	*p* value
Age group (years)			
18–30 (Ref)			
30–40	2.73 (0.83–8.99)	1.80 (0.48–6.79)	0.384
41–50	2.67 (0.82–8.64)	1.99 (0.55–7.23)	0.293
51–60	4.00 (1.22–13.17)	3.42 (0.91–12.88)	0.069
> 60	8.00 (2.29–27.90)	5.17 (1.27–21.09)	**0.022**
Residence			
Urban (Ref)			
Rural	1.57 (0.83–2.99)	2.03 (0.91–4.52)	0.082
BMI			
Normal (Ref)			
Underweight	2.18 (0.99–4.80)	2.12 (0.82–5.52)	0.122
Overweight	0.91 (0.34–2.40)	0.75 (0.25–2.28)	0.614
Obese	5.04 (1.16–21.92)	5.94 (1.01–34.95)	**0.049**
Diabetes duration			
< 5 years (Ref)			
> 5 years	2.85 (1.53–5.31)	2.25 (1.02–4.98)	**0.045**
Medication type			
Single oral (Ref)			
Combination oral	1.94 (1.01–3.70)	1.26 (0.55–2.91)	0.587
Insulin	0.77 (0.32–1.86)	0.69 (0.25–1.93)	0.476
Oral + insulin	1.55 (0.54–4.49)	1.21 (0.35–4.10)	0.764
Glycemic control			
Good (Ref)			
Poor	2.34 (1.32–4.14)	2.96 (1.48–5.91)	**0.002**
Neuropathy			
No (Ref)			
Yes	3.59 (1.99–6.49)	4.97 (2.48–9.98)	**< 0.001**

*Note:* Ref, reference category. Bold is *p* < 0.05.

Abbreviations: aOR, adjusted odds ratio; cOR, crude odds ratio.

## 4. Discussion

In this cohort of adults with DM attending MRRH, anemia affected 28.0% (69/246), indicating a clinically important comorbidity burden in routine diabetes care. This prevalence is strikingly similar to the global pooled prevalence of 27% among adults with type 2 diabetes and aligns with pooled estimates reported for Africa in meta‐analytic evidence, supporting the view that anemia is a frequent but often under‐recognized complication in diabetes care pathways [18]. Comparable facility‐based studies in the region report prevalence in a similar range, including 26.7% among diabetic adult outpatients in Northeast Ethiopia [19] and burdens reported among T2DM patients in Eastern Ethiopia [14]. Collectively, these patterns reinforce that integrating routine anemia screening into diabetes services is justified, particularly because anemia in diabetes is linked with worse renal and cardiovascular outcomes in observational evidence [20].

At the multivariable level, the independent associations observed—older age (> 60 years), neuropathy, longer diabetes duration (> 5 years), obesity, and poor glycemic control—suggest that anemia clusters with cumulative metabolic stress and diabetes complications rather than occurring randomly.

First, older age and longer duration of diabetes plausibly reflect longer exposure to microvascular injury and inflammatory burden, and they are repeatedly identified as predictors of anemia among diabetes populations in systematic review evidence [21]. Second, poor glycemic control may contribute through sustained oxidative stress and acceleration of microvascular complications, especially diabetic kidney disease, which reduces erythropoietin production and can precipitate anemia even before advanced renal failure becomes clinically obvious [22]. Third, obesity can drive iron‐restricted erythropoiesis via chronic low‐grade inflammation and hepcidin‐mediated impairment of iron absorption and iron mobilization [23].

Finally, diabetic neuropathy likely represents advanced disease and may also have a mechanistic link to anemia: autonomic neuropathy has been associated with a blunted erythropoietin response to anemia in T2DM patients without advanced renal failure, and lower hemoglobin has been associated with a higher prevalence of peripheral neuropathy in clinical datasets [24, 25]. Neuropathy may reflect more severe or long‐standing diabetes rather than directly causing anemia, and the cross‐sectional design also allows possible reverse causality. Therefore, the association should be interpreted as correlational, not causal. Taken together, these findings support a targeted clinic approach: prioritize anemia evaluation in older adults, those with long‐standing diabetes, poor control, and obesity, and neuropathy groups in whom anemia may signal underlying inflammatory and renal pathways.

Morphologically, anemia was predominantly normocytic (76.81%, 95% CI: 66.85%–86.77%), with smaller proportions of microcytic (13.04%, 95% CI: 5.10%–20.98%) and macrocytic anemia (10.14%, 95% CI: 3.02%–17.27%). This pattern is consistent with other diabetes cohorts where normocytic anemia is most common; for example, Ethiopia and Nigeria report normocytic predominance among anemic T2DM patients [15, 26]. The predominance of normocytic anemia supports contributions from anemia of chronic disease/inflammation and/or diabetic kidney disease, where hepcidin‐mediated iron restriction and reduced erythropoietin production (or hyporesponsiveness) are well‐described mechanisms [26]. The presence of microcytic anemia further suggests a concurrent burden of iron deficiency or iron‐restricted erythropoiesis, which is particularly relevant in low‐resource settings where inadequate dietary iron intake, chronic blood loss (including gastrointestinal causes), and inflammation‐mediated iron sequestration may coexist [23]. Although macrocytic anemia was least frequent, it remains clinically relevant because metformin use is associated with lower vitamin B12 levels and B12 deficiency in metformin‐treated T2DM patients, which can contribute to macrocytosis and anemia and may overlap with neuropathic symptoms [12, 27].

Overall, combining the prevalence, predictors, and morphology suggests a practical implication: anemia in diabetes care at MRRH is common and likely represents a mixed‐etiology phenotype (inflammation/renal pathways plus nutritional deficiencies). This supports routine hemoglobin screening with a stepwise evaluation guided by red cell indices, alongside focused assessment of renal function (creatinine/eGFR), iron status (ferritin ± transferrin saturation where available), and vitamin B12 (especially in metformin‐exposed patients), in line with mechanistic evidence in diabetes‐related anemia [26]. Future research should focus on longitudinal assessments to further elucidate the temporal relationship between diabetes progression, inflammatory markers, and the development of different anemia subtypes, as well as on the impact of therapeutic interventions on these parameters.

### 4.1. Strengths and Limitations

Strengths of this study include objective hematologic assessment using an automated hematology analyzer, description of red cell indices–based anemia patterns, and use of multivariable analysis to identify factors independently associated with anemia in adults with diabetes attending a regional referral hospital diabetic clinic. However, several limitations should be acknowledged. First, the cross‐sectional design limits causal inference. Second, renal function parameters such as serum creatinine, estimated glomerular filtration rate, and albuminuria were not measured, so subclinical diabetic kidney disease could not be assessed. Third, anemia morphology was classified using automated red cell indices, primarily MCV, without peripheral blood smear review or confirmatory iron, vitamin B12, folate, or inflammatory marker testing; therefore, etiology could not be established. Fourth, neuropathy was assessed clinically and not confirmed using a validated instrument or nerve conduction studies. Fifth, the obese subgroup was small, resulting in limited precision for that estimate. Finally, the facility‐based sample may limit generalizability beyond similar clinic populations.

## 5. Conclusion and Recommendations

Anemia was common among adults with DM attending MRRH, and normocytic anemia was the predominant red cell indices–based pattern. Older age, obesity, longer diabetes duration, poor glycemic control, and neuropathy were associated with higher odds of anemia. Routine anemia screening should be integrated into diabetes care, with further etiologic evaluation, particularly renal and micronutrient assessment, where feasible.

## Author Contributions

Feisal Dahir Kahie was the principal investigator, who formulated and designed the study and collected the data. Hanan Asad Hassan, Venance Emmanuel Mswelo, and Hamdi Mohamed Yusuf had significant contribution in data collection and data entry. Theoneste Hakizimana, Marie Pascaline Sabine Ishimwe, Elias Joseph Xwatsal, Abshir M. Hirsi, and Banturaki Amon analyzed the data and wrote the draft of the manuscript. Stella Nabirye, Banturaki Amon, Emmanuel Ifeanyi Obeagu, and Theoneste Hakizimana supervised the study and approved the manuscript. Theoneste Hakizimana, Feisal Dahir Kahie, and Marie Pascaline Sabine Ishimwe contributed to the drafting of the manuscript.

## Funding

No specific grant from public or nonprofit organizations funded this research.

## Disclosure

All the coauthors agreed to submit the final version of manuscript.

## Ethics Statement

Ethical approval was obtained from the Kampala International University Research Ethics Committee (Ref no: KIU‐2024‐399). Written informed consent was obtained from all participants before enrollment. Participation was voluntary, and confidentiality was maintained through the use of unique study identifiers and restricted access to the data. The study was conducted in accordance with the Declaration of Helsinki.

## Consent

Please see the Ethics Statement.

## Conflicts of Interest

The authors declare no conflicts of interest.

## Data Availability

The dataset that was utilized in this study is not publicly available due to ethical considerations. Upon reasonable request, the dataset used can be accessed with the permission of the corresponding author: Theoneste Hakizimana, Email: theonestehakizimana5@gmail.com.
